# Supplementary material for: Body size dependent dispersal influences stability in heterogeneous metacommunities
Source: Sci Rep. 2021 Aug 31;11:17410. doi: 10.1038/s41598-021-96629-5 (PMC8408130; doi:10.1038/s41598-021-96629-5)
Supplement: Supplementary file 1 — Supplementary Figures. [file 41598_2021_96629_MOESM1_ESM.pdf]

Supplementary Information: Body size dependent dispersal influences stability in heterogeneous metacommunities

Kurt E. Anderson[1,*] and Ashkaan K. Fahimipour[2,3]

[1]*Department of Evolution, Ecology, & Organismal Biology, University of California, Riverside, California, USA*
[2]*Department of Computer Science, University of California, Davis, California, USA*
[3]*Current Address: Institute of Marine Sciences, University of California, Santa Cruz, California, USA*
[*]*To whom correspondence should be addressed: kurt.anderson@ucr.edu*

Supplementary figures

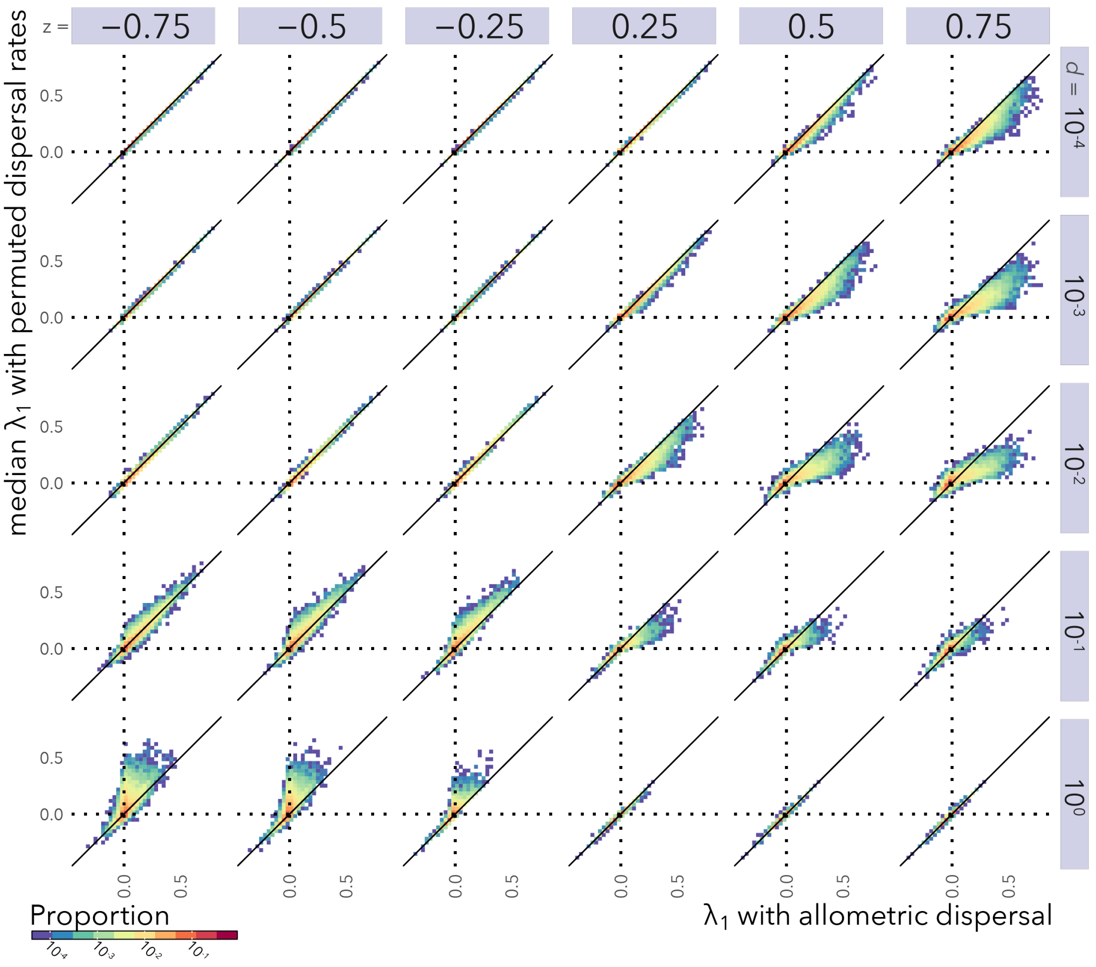

Figure S1: The effect of dispersal variation on metacommunity stability for all combinations of d and z (see Main Text). The metacommunity is stable when the real part of the leading eigenvalue of the metacommunity Jacobian $\lambda_1 < 0$. Allometric dispersal is defined in eq. 3 of the Main Text. Permuted dispersal refers to the metacommunity where the allometric dispersal rates for all species were randomly reassigned to new species. Each data point represents a unique metacommunity with allometric dispersal compared to 100 counterparts with dispersal rates randomly rearranged among species, and are shown with 1:1 lines. Points that lie above the 1:1 line represent cases where the median value of λ_1 for the permuted dispersal metacommunities are greater than the corresponding original metacommunity, indicating that the permuted metacommunities are typically less stable. Points that lie below the 1:1 line represent cases where the permuted metacommunities are typically more stable. Grey regions mark portions of the plot representing qualitative changes in stability where the real part of the leading eigenvalue λ_1 of the original metacommunity has a different sign than the median value of the eigenvalues of the comparable metacommunities with permuted dispersal.

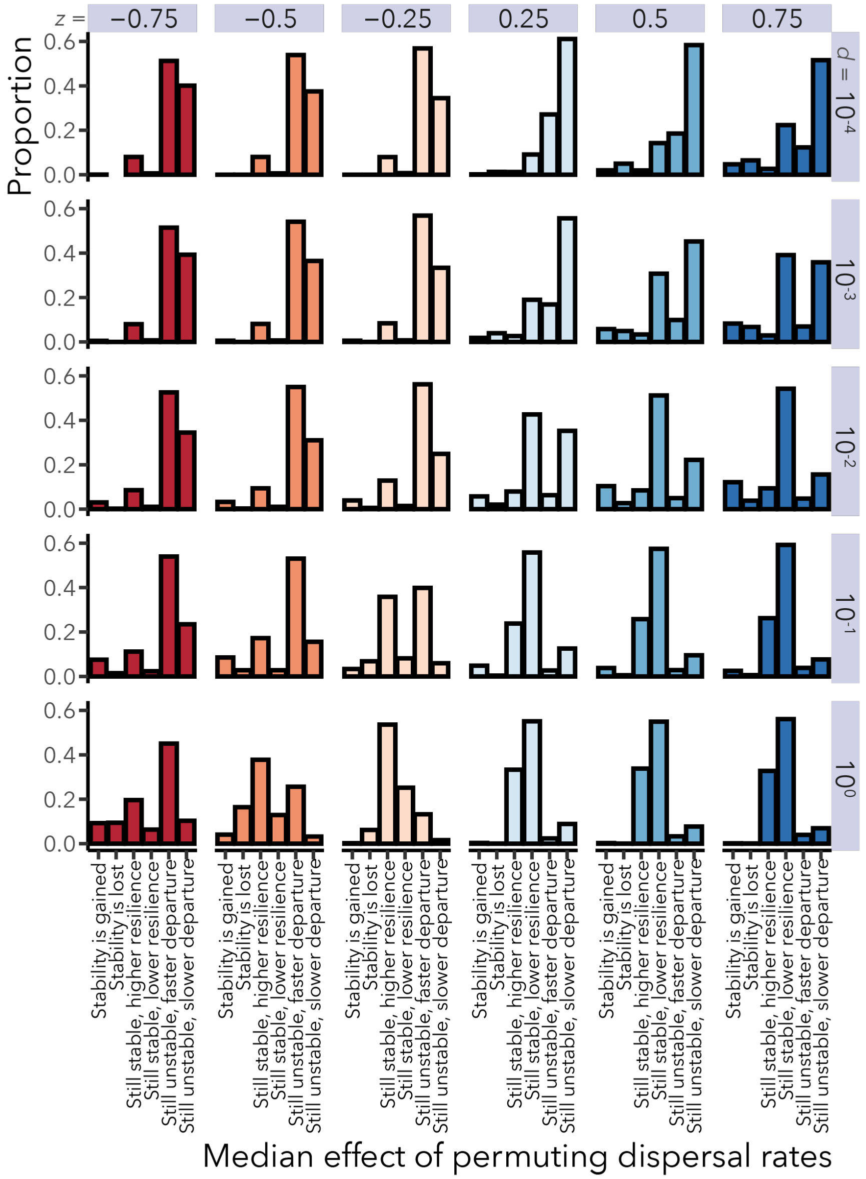

Figure S2: Eigenvalues λ_1 from Fig. S1 categorized by qualitative effects on stability for all combinations of d and z (see Main Text). Categories *Stability is gained* and *Stability is lost* correspond to cases where the median effect of permuting dispersal rates is a change in sign of λ_1. The remaining categories refer to cases where the magnitude, but not the sign, of the median λ_1 changes, leading to faster or slower departures from (or returns to) an unstable (or stable) steady state.
